# Supplementary material for: Learning curve and surgical outcome of robotic assisted colorectal surgery with ERAS program
Source: Sci Rep. 2022 Nov 29;12:20566. doi: 10.1038/s41598-022-24665-w (PMC9709162; doi:10.1038/s41598-022-24665-w)
Supplement: Supplementary file 2 — Supplementary Information 2. [file 41598_2022_24665_MOESM2_ESM.zip › RAL Raw data-Ñ[▒K/101/OP_ñw╝╨░O▒Kñσ.pdf]

正式摘要(電子簽章：0000022159113)

W52 / W52

臺中榮民總醫院

Operation Note

[redacted]

- 手術日期：2021/07/28
- 手術醫師：VS 林俊余，CR 林陳彥，R 陳映晴，R 林雋庭
- Anesthesia : GE
- Position : Lithotomy
- Aseptic agents : Betadine Aqua , Zephnan

Diagnosis

- Pre-operative diagnosis : Rectum ca s/p CCRT
- Post-operative diagnosis : s/p robotic total mesorectal excision (TME) + loop ileostomy

Surgical Indication

- For removing the primary tumor

Operation

- 1. Robotic (Xi) assisted TME + loop ileostomy
  2. Intraoperative colonoscopy : (-).
  3. High ligation (+) : At IMA root. IMV was also ligated.
  4. Take down from the splenic flexure : (-)
  5. Robotic EndoGIA (45-4.3) X4 + EEA (29mm) were applied to complete the end-to-end anastomosis
  6. Proximal cut end: > 10 cm, distal cut end: 0.5 cm
  7. A 12 Fr. penrose drainage tube was inserted from RLQ of abdomen (8mm trochar site) into anastomosis (Pelvic) area
  8. Loop ileostomy (About 15~20cm from IC valve) was done over LLQ of abdomen

Finding

- 1. The tumor was about 5cm FAV. Finger tip could reach the tumor.
  2. Moderately adhesion was noted over the pelvic area.
  3. No obvious ascites was presented.
  4. ICG test : 28s
  5. The proximal limb of the loop ileostomy could not be identified.

Procedure

- 1. General endotracheal anesthesia was induced and then the patient was placed in modified Trendelenburg lithotomy position.
  2. Preoperative antibiotics were given. A Foley catheter and nasogastric tube were placed.
  3. The abdomen was prepped and draped in standard sterile fashion.
  4. A transverse incision was made over the right side of the umbilicus and a 12 mm trocar was inserted into the peritoneal cavity by open method. Peumoperitoneum was established with CO2 inflation.
  5. Laparoscopy was inserted and then other two 8mm trocars were inserted over LUQ area of abdomen, also another one over RLQ area of abdomen. Finally, a 12mm trocar was inserted over RUQ area as an assist-port. The operative findings were mentioned as above. After the setting was completed, the laparoscopy was shifted to the LUQ 8mm trocar site instead.
  6. The sigmoid colon was held by grasper and the peritoneum overlying the mesentery was scored with electrocautery.
  7. The inferior mesentery artery was identified, ligated above the left colic artery with robotic clips, and transected. IMV was ligated via robotic clips and then also transected.

- 8. Dissection was done above the Gerota's fascia and toward the peritoneal attachments.
  9. The colon was mobilized from retroperitoneum overlying the sacral promontory and into the pelvis.

  10. Distal descending colon and s-colon were freely mobilized after the mesoclon was all dissected.
  11. The incision was then extended laterally and medially across the peritoneal reflection.
  12. The mesorectum was then mobilized using sharp dissection over the level of pelvic floor muscle. Then rectum was mobilized as possible.
  13. DRE was performed at the same time to make sure the desired resection line is adequate.
  14. The distal rectum was divided with robotic endoGIA (45-4.3) X4.
  15. A new incision was made over LLQ area of abdomen. We approached into the peritoneal cavity by open method. S-colon was pulled out.
  16. The proximal colon was then taken out for carefully inspection, and Indocyanine green agent was administered again from IV line, to make sure it has an excellent blood supply. The mesenteric fat was gently cleared from the terminal 2-3 mm of the bowel ends. (# The result of the testing time was 28 seconds)
  17. The field was isolated with moist pad and the bowel opened and sized. It was determined that a 29 mm circular stapler could be accommodated. A purse string suture of 2-0 prolene was placed. The tumor-containing tissue was removed. Then the anvil was inserted, followed by the purse string suture. The colon was then put back to the abdominal cavity.
  18. Pneumoperitoneum was constructed again.
  19. The anal canal was irrigated with distil water and then the circular stapler was introduced through the anus and pierced through the upper area (The ventral site of the body) of the stapler line.
  20. The proximal bowel with anvil in place was engaged with the circular stapler and the stapler was then fired. Two complete donuts were obtained and the distal one was submitted to pathology for margin confirmation.
  21. The anastomosis was tested for leaks with irrigation solution and air introduced through the rectum and was found to be patent and intact.
  22. A 12 Fr. penrose drainage tube was inserted from RLQ of abdomen (8mm trochar site) into anastmosis (Pelvic) area.
  23. The fascia of the 8mm and the 12mm trochar sites were all closed with interrupted sutures via 1-O Vicryl. The size of the LLQ extended wound was also reduced via applying one figure of eight suture via 1-O Vicryl.
  24. The desired ileum site (About 15~20cm from IC valve) was selected for further ostomy construction.
  25. The LLQ extended wound area was selected as the site for loop ileostomy.
  26. The ileum was retracted from the desired ostomy site. A window was created under the ileum by dividing the mesentery adjacent to the bowel wall with electrical cautery carefully, with a plastic bridge in placed.
  27. Massive N/S was applied to irrigate the wound.
  28. The wound was further closed with interrupted sutures via 3-O Nylon, then covered with Medipore.
  29. Moist pads were placed and the ileum was opened. Succus and air were aspirated.
  30. The ileostomy was matured with multiple interrupted mucocutaneous suture via 4-0 Monocryl.
  31. The ileostomy was well formed and viable. An ostomy bag was placed.
  32. The patient tolerated the procedure well and was brought to the POR in stable condition.
- Estimated blood loss: minimum
- 器械數量: 正確
- 紗布數量: 正確

Image

- PAINT
  - abdomen

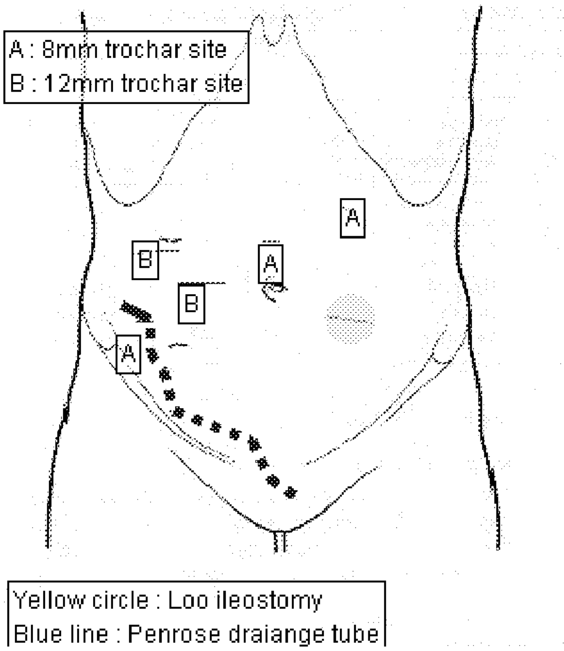

- colon & rectum

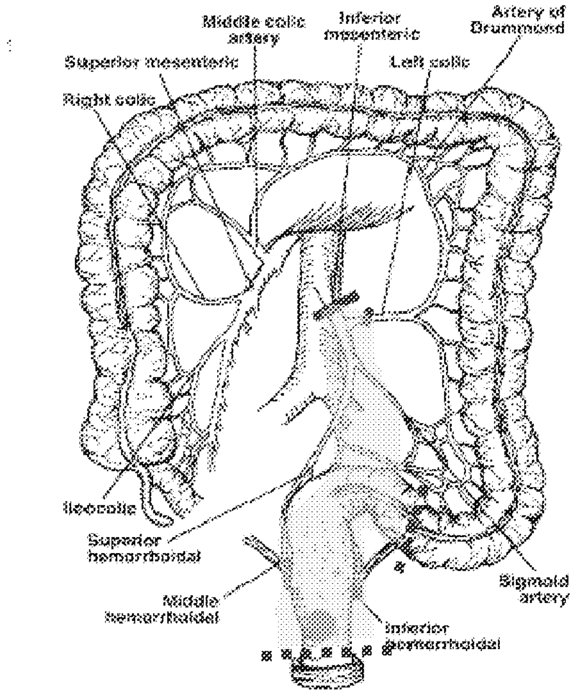

- The resected specimen

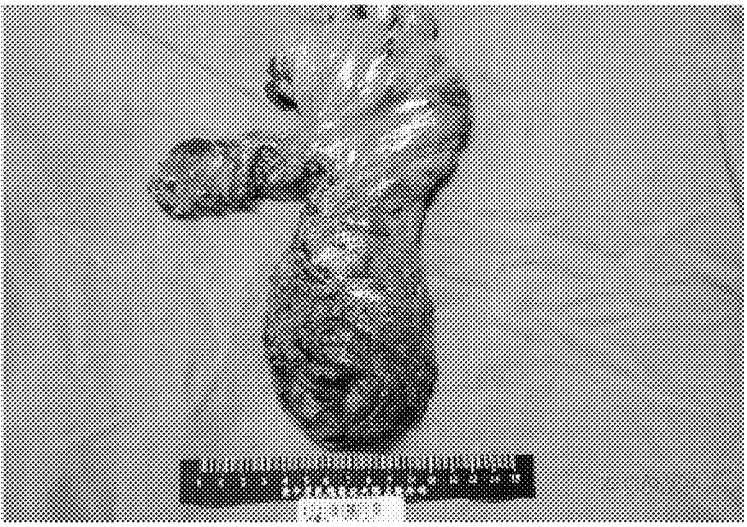

The tumor could be clearly seen

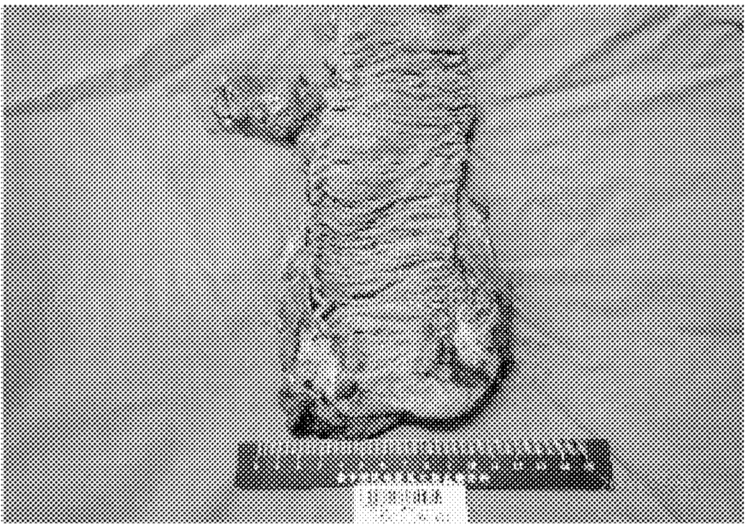

The necrotic tumor

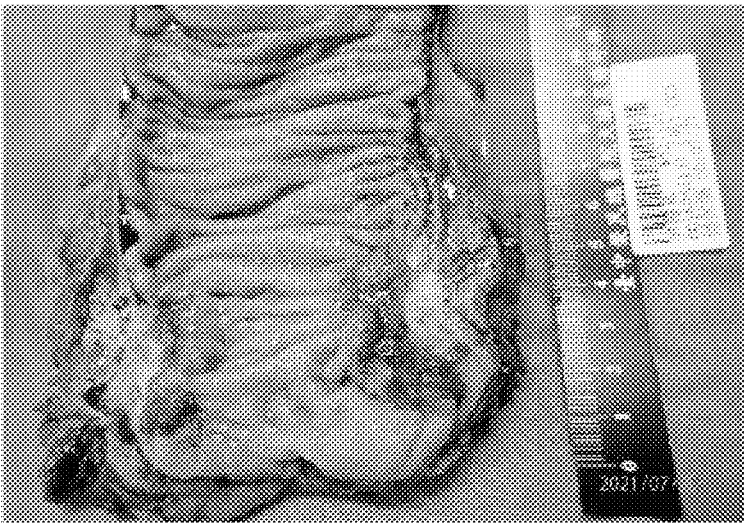

主治 2222H林俊余 / 醫師 2301H林陳彥

正式摘要(電子簽章：**000000221591113)**

W52 / W52

doctorRank:B
